# Relationship between 25-hydroxyvitamin D and IGF1: a cross-sectional study of the Third National Health and Nutrition Examination Survey participants

**DOI:** 10.1186/s41043-023-00374-6

**Published:** 2023-04-18

**Authors:** Wei Li, Tao Yu

**Affiliations:** 1 Department of Child Health Care, West China Second University Hospital, Sichuan University, No. 20, Section 3, Renmin South Road, Wuhou District, Chengdu, 610044 Sichuan China; 2 Key Laboratory of Birth Defects and Related Diseases of Women and Children (Sichuan University), Ministry of Education, No. 20, Section 3, Renmin South Road, Wuhou District, Chengdu, 610044 Sichuan China

**Keywords:** Bone, Insulin-like growth factor, NHANES, Vitamin D

## Abstract

**Background:**

25-Hydroxyvitamin D (25OHD) and insulin-like growth factor 1 (IGF1) are crucial for bone health. Some studies have shown that they interact, whereas others have indicated no association. However, it remains inconclusive whether the interaction between the two is dose dependent. Herein, we explored the relationship between 25OHD and IGF1 by conducting a cross-sectional study.

**Methods:**

This study involved 6,046 individuals from the Third National Health and Nutrition Examination Survey (NHANES III). The dependent and independent variables were IGF1 and 25OHD levels, respectively. The covariates included age, sex, race, BMI, exercise, smoking behavior, alcohol intake, diabetes, and serum calcium level. Multiple linear regression and generalized additive model were employed to analyze the relationship between 25OHD and IGF1. Interaction and hierarchical analyses were also performed.

**Results:**

The 25OHD and IGF1 levels positively correlated after adjusting for covariates (*β* = 0.16, 95% CI: 0.04–0.29, *P* = 0.0103). Smooth curve fitting demonstrated a curvilinear relationship. When the 25OHD level was < 75 nmol/L, a positive correlation (*β* = 0.43, 95% CI: 0.25–0.62, *P* < 0.0001) was observed. When the 25OHD level was > 75 nmol/L, a negative correlation was observed (*β* = −0.53, 95% CI: −0.90 to −0.15, *P* = 0.0057).

**Conclusion:**

This study demonstrated a nonlinear relationship between 25OHD and IGF1. It suggests that keeping the 25OHD level within a specific range may be more conducive to bone health. Additionally, when IGF1 is used to evaluate the efficacy and safety of recombinant human growth hormone (rhGH) in growth hormone deficiency treatment, the effect of 25OHD on the actual IGF1 level should be taken into account.

## Background

Vitamin D is crucial for mineral metabolism and bone health, and its deficiency can lead to various diseases including osteomalacia and rickets [1, 2]. Growth in children is mainly regulated by growth hormones, and insulin-like growth factor 1 (IGF1), a differentiation and growth factor, is closely related to the height-promoting growth hormones. Growth hormones and IGF1 also play essential roles in adult bone transformation [3–5]. As both IGF1 and vitamin D are critical for bone growth and metabolism, they may interact rather than function independently.

Vitamin D can promote the expression of IGF1 and IGF-binding protein 3 genes in the liver and bone tissue [6–8]. Vitamin D receptor knockout mice have a considerably lower serum IGF1 level than control group mice (approximately 29%) [9]. According to several clinical studies, vitamin D and IGF1 levels are positively correlated [10–14]. Some studies have reported that vitamin D and IGF1 cooperate to increase the height of children with short stature or rickets [15, 16]. However, the nature of the dose–response relationship between vitamin D and IGF1 remains controversial. Some studies suggest that vitamin D and IGF1 have a linear relationship, whereas others suggest a saturation effect or a negative feedback effect [13, 14]. Furthermore, doubts remain as to whether any relationship exists between the two. A randomly controlled experiment conducted by Trummer et al. revealed no difference in the IGF1 level between vitamin D-supplemented and placebo groups [17]. Meshkini’s meta-analysis also revealed that IGF1 level was unaffected by vitamin D supplementation [18].

The predominant form of vitamin D in humans is 25OHD. Given the above contradictory findings, herein, we explored the relationship between 25OHD and IGF1 by conducting a cross-sectional study using the data of the Third National Health and Nutrition Examination Survey (NHANES III), an American public database.

## Materials and methods

### Study population

**Fig. 1:**
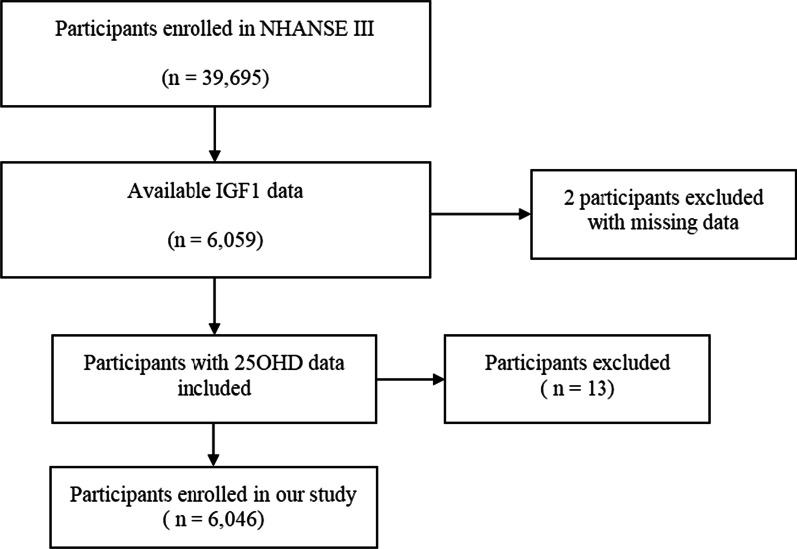
The flowchart of inclusion/exclusion criteria

The NHANES (https://www.cdc.gov/nchs/nhanes/index.htm), which assesses the lifestyle, health, and nutritional status of non-hospitalized Americans through complex multi-stage stratified cluster probability sampling, provided the data for our study. The data of participants of the NHANES III, conducted between 1988 and 1994, were used. Detailed data of the design and process of the NHANES are publicly available online. A total of 39,695 participants were enrolled in NHANSE III, of which 6,061 were tested for IGF1 and 2 had missing data. Of the remaining 6,059 participants, 13 lacked 25OHD data, so we finally included 6,046 participants for study (Fig. 1). Since the public can obtain all NHANES III data for free online (https://www.cdc.gov/nchs/nhanes/index.htm), the study was not subject to review by the institutional review board.

### Variables

The independent and dependent variables in our study were 25OHD and IGF1 levels, respectively. Vitamin D status was evaluated using the 25OHD level. The covariates were age, sex, race, BMI, exercise, smoking behavior, alcohol intake, diabetes, and serum calcium level. The serum level of 25OHD was initially detected using the DiaSorin RIA kit (Stillwater, MN, USA), and then converted using regression to equivalent standardized liquid chromatography-tandem mass spectrometry measurements. IGF1 level was determined using standard laboratory protocols from Diagnostic Systems Laboratories Inc. (Webster, TX, USA). Continuous variables included age, BMI, 25OHD, IGF1, and serum calcium level, whereas categorical variables included sex, race, alcohol intake, smoking behavior, exercise, and diabetes. The detailed investigation process can be found at http://www.cdc.gov/nchs/nhanes/index.htm.

### Statistical analysis

For all analyses, we considered the sample weight of the NHANES. First, the missing covariate data were processed, and thereafter, the missing continuous data were supplemented with the mean, whereas missing data for categorical variables were treated as a new classification set. We described the baseline characteristics of all study populations based on the tertiles of 25OHD levels. The continuous data are presented as mean ± SD, and the *P* value was calculated using the weighted linear regression model. Categorical data are presented in terms of percentage, and the *P* value was calculated using the Chi-square test. Based on the recommendation of the STROBE statement [19], three linear regression models (unadjusted, partially adjusted, and fully adjusted) were utilized to determine the effect of 25OHD on IGF1. We performed a sensitivity analysis by transforming the continuous variable 25OHD into a categorical variable to measure the *P* value for trend. A generalized additive model was employed to verify the curvilinear association between 25OHD and IGF1 levels. We then applied segmented regression, which fit each interval with a separate line segment. To ascertain whether a critical value existed, we employed the log-likelihood ratio test to compare the linear model with the nonlinear fitting model. If the *P* value was < 0.05, the difference between the linear and nonlinear fitting models was considered significant. Based on the model providing the maximum likelihood, the inflection point joining the segments was measured using a two-step recursive algorithm. We studied the effects of different subgroups on the relationship between 25OHD and IGF1 using interaction and stratification analyses. Each subgroup was adjusted for all the affected elements, except the stratification factor itself. For data analysis, R software (http://www.R-project.org, R Foundation) and Empowerstats (http://www.empowerstats.net/analysis/index4.php, X&Y Solutions, Inc., Boston, MA, USA) were used. Statistical significance was defined as a *P* value < 0.05.

## Results

### Baseline characteristics of the participants

The participants were 20–90 years old, with an average age of 46.8 ± 18.8 years. Of the total participants, 45.3% and 54.7% were male and female, respectively. The mean IGF1 and 25OHD levels were 256.2 ng/mL and 54.4 nmol/L, respectively. According to the 25OHD level, the participants were classified into tertiles (< 42.8, 42.8–62.6, and > 62.6). The numbers assigned to the three groups were 1995, 2028, and 2023, respectively. The baseline characteristics are represented in Table 1. The group with the highest 25OHD level had a higher serum IGF1 level and lower age and BMI value than the other groups. The serum calcium level was significantly different among the groups.

**Table 1:** Baseline characteristics of 6,046 subjects, based on 25OHD tertiles

25OHD, nmol/L	T1(< 42.80)	T2(42.80–62.60)	T3(> 62.60)	*P* value
Number	1995	2028	2023	
IGF1, mean ± SD, ng/mL	258.27 ± 103.37	258.82 ± 100.17	286.82 ± 107.65	< 0.0001
Age, mean ± SD, year	44.68 ± 16.43	45.75 ± 16.29	41.22 ± 16.10	< 0.0001
BMI, mean ± SD, kg/m^2^	28.09 ± 6.50	27.11 ± 5.42	25.62 ± 5.12	< 0.0001
Serum calcium, mean ± SD, mmol/L	1.24 ± 0.04	1.24 ± 0.05	1.24 ± 0.03	0.0318
Gender, %				< 0.0001
Male	33.62	45.85	52.37	
Female	66.38	54.15	47.63	
Race, %				< 0.0001
Non-Hispanic white	49.03	74.71	89.15	
Non-Hispanic black	32.4	9.7	2.51	
Mexican–American	7.94	6.42	3.32	
Other	10.63	9.17	5.02	
Exercise, %				< 0.0001
Mild	53.48	49	43.9	
Moderate	46.15	50.65	55.97	
High	0.29	0.19	0.06	
NA	0.08	0.17	0.07	
Smoking behavior, %				< 0.0001
Current	31.91	27.01	25.8	
None	46.36	46.31	46.3	
Past	21.73	26.68	27.91	
Alcohol intake, %				< 0.0001
Up to one day a week	77.07	73.1	70.34	
One to three days a week	9.53	12.77	17.51	
Three to six days a week	9.86	11.66	10.32	
Daily	0.1	2.47	0.03	
NA	3.44		1.79	
Diabetes, %				< 0.0001
Yes	6.45	3.41	3.15	
No	93.42	96.51	96.61	
NA	0.12	0.08	0.24	

Abbreviations: 25-hydroxyvitamin D (25OHD), insulin-like growth factor 1 (IGF1), body mass index (BMI), not available (NA)

### Association between IGF1 and covariates as determined using the univariate analysis

The outcomes of the univariate analysis with IGF1 are displayed in Table 2. The results indicate the following:

1. The 25OHD level was positively correlated with the IGF1 level.
2. Age and BMI were negatively correlated with the IGF1 level.
3. The IGF1 level was lower in women than in men.
4. The IGF1 level in non-Hispanic whites was lower than that in non-Hispanic blacks but higher than that in Mexican–Americans.
5. Smokers had a lower IGF1 level than non-smokers.
6. High drinkers had lower IGF1 levels than low drinkers.
7. The levels of serum calcium and IGF1 were not significantly correlated.

**Table 2:** Univariate analysis of IGF1

Variable	Statistics	β (95% CI)	*P* value
25OHD, nmol/L	54.37 ± 20.66	0.16 (0.04, 0.29)	0.0103
Age, year	46.76 ± 18.77	−3.04 (−3.19, −2.89)	< 0.0001
BMI, kg/m^2^	27.23 ± 5.74	−2.21 (−2.64, −1.79)	< 0.0001
Serum calcium, mmol/L	1.24 ± 0.04	−6.29 (−61.22, 48.65)	0.8225
Gender, *n* (%)			
Male	2740 (45.32%)	Ref	
Female	3306 (54.68%)	−25.43 (−30.20, −20.67)	< 0.0001
Race, *n* (%)			
Non-Hispanic white	2552 (42.21%)	Ref	
Non-Hispanic black	1636 (27.06%)	9.62 (1.56, 17.69)	0.0194
Mexican–American	1610 (26.63%)	−35.82 (−46.34, −25.29)	< 0.0001
Other	248 (4.10%)	−3.11 (−12.06, 5.84)	0.4963
Exercise, *n* (%)			
Mild	3277 (54.20%)	Ref	
Moderate	2744 (45.39%)	2.33 (−2.28, 6.95)	0.3214
High	19 (0.31%)	−47.33 (−106.50, 11.83)	0.1169
NA	6 (0.10%)	−62.52 (−132.71, 7.67)	0.0809
Smoking behavior, *n* (%)			
Current	1511 (24.99%)	Ref	
None	3045 (50.36%)	9.50 (3.90, 15.11)	0.0009
Past	1490 (24.64%)	6.14 (−0.26, 12.53)	0.0601
Alcohol intake, *n* (%)			
Up to one day a week	4579 (75.74%)	Ref	
One to three days a week	724 (11.97%)	−1.12 (−7.80, 5.56)	0.7421
Three to six days a week	532 (8.80%)	−15.99 (−23.65, −8.33)	< 0.0001
Daily	3 (0.05%)	−33.21 (−152.40, 85.98)	0.585
NA	208 (3.44%)	−12.30 (−27.48, 2.88)	0.1122
Diabetes, *n* (%)			
Yes	388 (6.42%)	Ref	
No	5652 (93.48%)	−10.88 (−22.90, 1.14)	0.0762
NA	6 (0.10%)	−27.75 (−85.27, 29.77)	0.3445

All analyses were adjusted for age, sex, race, exercise, smoking behavior, alcohol intake, diabetes, serum calcium level, BMI, and 25OHD level

Abbreviations: 25-hydroxyvitamin D (25OHD), insulin-like growth factor 1 (IGF1), body mass index (BMI), not available (NA)

### Association between the 25OHD and IGF1 levels

The association between the 25OHD and IGF1 levels was analyzed using a multiple linear regression model (Table 3), which contained three models. The results of the original unadjusted model (model I) showed that the IGF1 level positively correlated with the 25OHD level (*β* = 0.68, 95% CI: 0.56–0.81, *P* < 0.0001). Model II also revealed a positive correlation between the variables (*β* = 0.30, 95% CI: 0.18–0.42, *P* < 0.0001). In model III, the IGF1 level remained positively correlated with the 25OHD level (*β* = 0.16, 95% CI: 0.04–0.29, *P* = 0.0103), but the effect value decreased. When the 25OHD level increased by 1 nmol/L, the IGF1 level increased by 0.16 ng/mL. We then performed a sensitivity analysis using the 25OHD level as a classification variable. In model III, compared to the T1 IGF1 level, the T3 IGF1 level increased by 8.41 ng/mL. The non-isometric changes in the effect values of the three groups indicated a possible nonlinear association between 25OHD and IGF1.

**Table 3:** Association between 25OHD and IGF1 in different models

	Model I (*β*, 95% CI, *P*)	Model II (*β*, 95% CI, *P*)	Model III (*β*, 95% CI, *P*)
25OHD	0.68 (0.56, 0.81) < 0.0001	0.30 (0.18, 0.42) < 0.0001	0.16 (0.04, 0.29) 0.0103
25OHD (tertiles)			
Tertile 1	Reference	Reference	Reference
Tertile 2	0.55 (−6.94, 8.05) 0.8847	2.68 (−4.12, 9.49) 0.4396	0.19 (−6.58, 6.96) 0.9566
Tertile 3	28.55 (21.53, 35.56) < 0.0001	14.62 (7.86, 21.38) < 0.0001	8.41 (1.60, 15.21) 0.0155
*P* for trend	< 0.001	< 0.001	0.004

Model I: no adjustments

Model I: adjusted for age, gender, and race

Model III: adjusted for age, sex, race, exercise, smoking behavior, alcohol intake, diabetes, serum calcium, and BMI

Abbreviations: 25-hydroxyvitamin D (25OHD), insulin-like growth factor 1 (IGF1), body mass index (BMI)

### Nonlinear relationship between 25OHD and IGF1

**Fig. 2:**
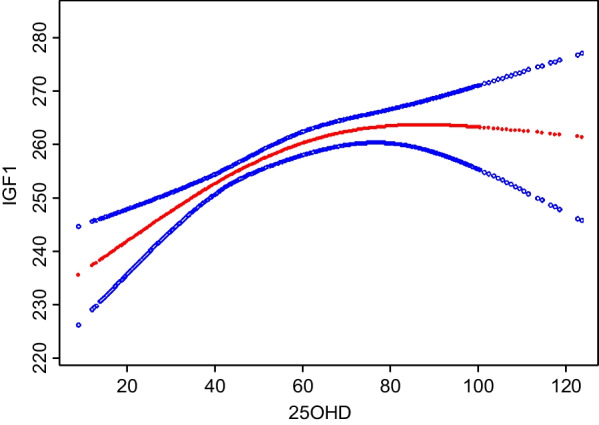
Nonlinear relationship between 25OHD and IGF1. 25OHD, 25-hydroxyvitamin D; IGF1, insulin-like growth factor

A generalized additive model was employed for further analysis of the potential nonlinear relationship between 25OHD and IGF1. The smooth curve produced using the generalized additive model demonstrated that after adjusting for age, sex, race, BMI, exercise, smoking behavior, alcohol intake, diabetes, and serum calcium level, the relationship between 25OHD and IGF1 was nonlinear, with an inverted U shape (Fig. 2). Table 4 presents the linear and nonlinear fitting models used to verify the relationship between 25OHD and IGF1 levels (*P* < 0.001). In the segmented regression model (nonlinear fitting model), the inflection point of 25OHD level was 75 nmol/L. 25OHD and IGF1 had a positive association on the left side of the inflection point (*β* = 0.43, 95% CI: 0.25–0.62, *P* < 0.0001) and a negative association on the right side (*β* = −0.53, 95% CI: −0.90 to −0.15, *P* = 0.0057).

**Table 4:** Comparison of the linear and nonlinear fitting models

	IGF1 (*β* 95% CI)	*P* value
Fitting model by standard linear regression	0.16 (0.04, 0.29)	0.0103
Fitting model by two-piecewise linear regression inflection point of 25OHD	75	
≤ 75	0.43 (0.25, 0.62)	< 0.0001
> 75	−0.53 (−0.90, −0.15)	0.0057
*P* for log-likelihood ratio test		< 0.001

Age, sex, race, exercise, smoking behavior, alcohol intake, diabetes, serum calcium level, and BMI were adjusted for in the model

Abbreviation: insulin-like growth factor 1 (IGF1), 25-hydroxyvitamin D (25OHD), body mass index (BMI)

### Subgroup analysis

**Fig. 3:**
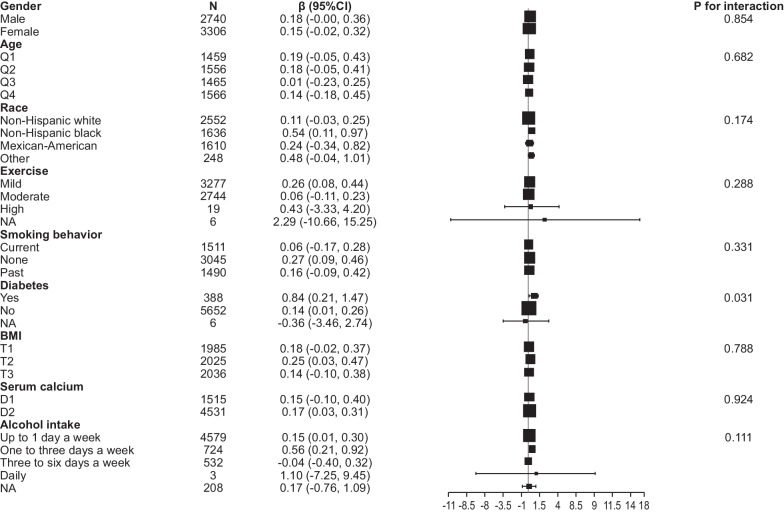
Interaction and hierarchical analyses of the effect of 25OHD on IGF1. 25OHD, 25-hydroxyvitamin D; IGF1, insulin-like growth factor

We investigated the effect of other covariables on the relationship between 25OHD and IGF1 through interaction and hierarchical analyses. To analyze the trend of effect values among the covariables, we used age, sex, race, BMI, exercise, smoking behavior, alcohol intake, diabetes, and serum calcium level as stratified variables (Fig. 3). The results showed a highly consistent pattern. The directions of the effect values were almost the same, indicating that 25OHD and IGF1 levels were positively correlated in each subgroup. 25OHD had a stronger effect on IGF1 (*P* for interaction = 0.031) in patients with diabetes than in those without diabetes. However, in the subgroups adjusted for age, sex, race, BMI, exercise, smoking behavior, alcohol intake, and serum calcium level, there was a consistent association between 25OHD and IGF1 (*P* for interaction > 0.05).

## Discussion

As 25OHD and IGF1 are important for maintaining bone health, the main purpose of our study was to explore whether there is an association between the two, and if so, how they are associated. Accordingly, a cross-sectional study of a nationally representative non-hospitalized population in the USA was performed. After considering the influencing factors, we found that there was an independent correlation between 25OHD and IGF1. In addition, we found that there was a nonlinear relationship between them. When the 25OHD level increased to 75 nmol/L, the relationship between 25OHD and IGF1 changed from positive to negative. The subgroup analysis helped us better understand the trend of the association between 25OHD and IGF1 in different populations. 

Our study also found that high drinkers had lower IGF1 levels than low drinkers. Previous studies found that the expression of IGF1 in the liver, skeletal muscle, or brain of mice exposed to alcohol significantly decreased. The specific mechanism may be related to the extensive damage to the signal pathway of insulin-like growth factor caused by alcohol [20–23]. Furthermore, the result of the subgroup analysis showed that 25OHD had a stronger effect on IGF1 in patients with diabetes than in those without diabetes. Many studies have found that both vitamin D and IGF1 play a vital role in the regulation of glucose metabolism. They are related to the improvement of glucose homeostasis and insulin sensitivity in diabetes [24–27]. Therefore, there may be some interaction between the two in diabetes. However, limited research is available on this; further studies are needed to explore the extraosseous interaction between vitamin D and IGF1.

The relationships between vitamin D and IGF1 have been studied in adults, children, and healthy or short stature groups, but the association between these factors remains controversial. Ameri et al. found that vitamin D level positively correlates with IGF1 level in adults with growth hormone deficiency. Furthermore, circulating IGF1 level significantly increased with increased vitamin D3 supplementation. Another interesting finding of this study was that vitamin D level was negatively correlated with the dose of recombinant human growth hormone (rhGH) [10]. A randomized controlled trial in healthy children revealed that individuals who received higher vitamin D3 supplementation doses had higher serum IGF1 levels [11]. These studies indicate that the effect of vitamin D on IGF1 is dose dependent.

Conversely, Hyppönen et al. found a saturation effect, whereby an increasing 25OHD level causes the IGF1 level to initially increase linearly. However, when the 25OHD level reaches 75–85 nmol/L, the effect stabilizes and the IGF1 level plateaued [13]. Concurrently, Kord-Varkaneh et al. suggested that there may be a negative feedback effect between vitamin D and IGF1. Their meta-analysis showed that serum vitamin D and IGF1 levels have an initial positive dose-dependent relationship. Nevertheless, vitamin D is negatively associated with IGF1 when its supplemented dose is more than 1000 IU/d and the supplementation duration is more than 12 weeks [14]. Our results are in agreement with those of Kord-Varkaneh et al. [14]. The smoothing curve generated with the generalized additive model showed that after adjusting for confounding factors, the IGF1 and 25OHD levels had an inverted U-shaped association (Fig. 2). When 25OHD level is under 75 nmol/L, it positively correlated with the IGF1 level. Contrastingly, when 25OHD level is over 75 nmol/L, a negative correlation was detected. According to the Global Consensus Recommendations on Prevention and Management of Nutritional Rickets, vitamin D status was classified according to 25OHD levels as follows: (1) Deficiency (< 30 nmol/L); (2) Insufficiency (30–50 nmol/L); (3) Sufficiency (>50 nmol/L); (4) Toxicity (>250 nmol/L) [28]. Our research results show that when the 25OHD level increased to 75 nmol/L, the relationship between 25OHD and IGF1 changed from positive to negative. That is, the 25OHD level increased by 1 nmol/L, whereas IGF1 level decreased by 0.53 ng/mL. Therefore, keeping 25OHD level at 50–75 nmol/L may be more beneficial for maintaining bone health and IGF1 level. However, the specific mechanism leading to this dose–response relationship has not been studied; hence, additional research is needed in the future to elucidate and validate the association.

Our findings suggest that within a specific range, vitamin D is positively associated with IGF1, but beyond a particular value, vitamin D may have an inhibitory effect on IGF1. Therefore, identifying an ideal range of vitamin D level may be helpful for maintaining IGF1 levels and promoting bone health. Furthermore, the effect of vitamin D must be considered when evaluating IGF1 level during rhGH therapy in growth hormone deficiency.

In addition, it is noteworthy whether vitamin D plays a role in the treatment of short stature with rhGH. The growth hormone-insulin-like growth factor 1 axis is important for linear growth in children and bone health in adulthood. Growth hormone is secreted by the pituitary gland and plays a role in promoting linear growth, mainly through IGF1. Our results are consistent with the previous studies indicating a possible relationship between vitamin D and IGF1. Several studies found that vitamin D is positively correlated with growth hormone peak [29, 30]. Ameri’s research even found that vitamin D level was negatively correlated with the dose of rhGH [10]. Therefore, future research could examine the long-term effects of vitamin D and rhGH on the growth parameters with short stature and if vitamin D supplements can reduce rhGH doses.

The following are some advantages of our study; to the best of our knowledge, this is the first in-depth research on the specific effect of 25OHD on IGF1. Second, a nationwide probability sample of the American population was selected by employing data from the NHANES; hence, strict quality control measures were applied. Third, we adjusted for potential confounding factors and performed a sensitivity analysis and hierarchical interactive analysis, whereby these methods can reduce the chances of introducing bias during data analysis; moreover, their application supports the conclusion that our research results are stable. Additionally, we used a generalized additive model to analyze and determine the association between 25OHD and IGF1. We also utilized a segmented regression model to calculate the specific inflection point of 25OHD, these implementations gave our findings higher clinical value and significance compared to similar studies performed previously.

Nevertheless, our study also had some limitations. Evidently, the observational nature is the main limitation as a cross-sectional design alone is insufficient to examine the causal association between 25OHD and IGF1. In addition, selection bias, information bias, recall bias, and others are also considered its limitations; hence, more randomized controlled trials are required. Second, the study group included only adults; hence, further research is warranted to determine the interaction between 25OHD and IGF1 in children. Finally, other confounding factors such as diet, vitamin D supplementation, and the use of rhGH might not have been included, ultimately limiting the robustness of the results shown here.

In conclusion, our findings demonstrated a nonlinear relationship between 25OHD and IGF1 and the significance of our results toward maintenance of bone health warrants further comprehensive research that includes prospective randomized controlled studies of different populations and identification of additional confounding factors.

## Data Availability

The datasets generated and/or analyzed during the current study are available in the NHANES repository, https://www.cdc.gov/nchs/nhanes/index.htm.
